# Hummingbirds modify their routes to avoid a poor location

**DOI:** 10.3758/s13420-021-00476-3

**Published:** 2021-08-02

**Authors:** Maria C. Tello-Ramos, T. Andrew Hurly, Mabel Barclay, Susan D. Healy

**Affiliations:** 1 School of Biology, University of St Andrews, St Andrews, UK; 2 Department of Biological Sciences, University of Lethbridge, Alberta, Canada

**Keywords:** traplining, route optimization, recursive movements, spatial cognition, hummingbirds

## Abstract

Traplining, when animals repeat the order in which they visit a number of locations, is taxonomically widespread, but little is known about which factors influence the routes that animals follow. For example, as the quality of rewarding locations changes over time, foragers are expected to update their traplines, either to prioritize locations where the reward increases or to avoid locations that have ceased to be profitable. Here, we tested how traplining wild hummingbirds responded to increases or to decreases in the sucrose concentration of one of the flowers on their trapline. Hummingbirds did not change their trapline to visit the flower with the increased reward first, but by changing the order in which they visited flowers, they avoided a flower that contained a decreased reward. Depending on where along the trapline the reduced-content flower occurred, hummingbirds either changed the origin of their trapline or changed the direction in which they flew around their trapline. It may be that this asymmetric modification of foraging traplines is especially noticeable in risk-averse foragers, such as these territorial hummingbirds.

**Supplementary Information:**

The online version contains supplementary material available at 10.3758/s13420-021-00476-3.

When animals return to the same locations again and again, they often develop a relatively short, optimal route around those locations (called traplining; Janzen, 1971). To do this, animals first have to learn the locations of the resources and the distances and directions between them. They may then compare the distances of different routes that connect all the locations to reach the shorter, more optimal route(s). This was shown to be the case when bumblebees (*Bombus terrestris*), which were tracked with harmonic radar, flew shorter and shorter routes around five feeding stations as they became more experienced (Lihoreau et al., 2012). The task of estimating the shortest route connecting several locations is analogous to the travelling salesperson problem (TSP), where an individual has to find the optimal (shortest) route around several locations before returning to the starting point (Schrijver, 2005). Even when there are only a few places to visit (e.g., five locations), the only known mathematical method for solving the problem is to calculate all possible routes (N!, which for five locations would be 120 different sequences) and then to compare each of the total distances for each sequence before selecting the shortest. As the number of locations included in a sequence increments, the task becomes increasingly difficult and time-consuming (Lawler et al., 1995). Animals are therefore expected to use simpler rules or heuristics of different types depending on the layout of the resources (Reynolds et al., 2013).

Regardless of the complexity of this task, a wide range of animals typically arrive at the shortest route connecting several locations (pigeons, *Columba livia,* Baron et al., 2015; rats, *Rattus norvegicus,* humans, *Homo sapiens sapiens,* Blaser & Ginchansky, 2012; bumblebees, *Bombus terrestris,* Lihoreau et al., 2010; Lihoreau et al., 2013; Ohashi et al., 2006; honeybees, *Apis mellifera,* Buatois & Lihoreau, 2016; butterflies, Gilbert, 1975; rufous hummingbirds, *Selasphorus rufus,* Tello-Ramos et al., 2015, 2019; vervet monkeys, *Chlorocebus pygerythrus,* Cramer & Gallistel, 1997). Once an animal arrives at the shortest route, it is likely to repeat it multiple times. What type of information is used when remembering and returning to a location can vary. For example, when resources are constant in space and time, desert ants (*Cataglyphis fortis*) take direct paths from the nest to a known resource by using path integration (Collett et al., 1999), while displaced honeybees combine path integration information and the location of landmarks to make shortcuts when homing or travelling to a feeder (Menzel et al., 2005). How animals update their routes between multiple locations when the resources change is less well understood.

Traplining produces movement patterns that should be subject to changes depending on the complexity and stability of the environment. If the environment is stable, pairwise comparisons between sequential routes might allow animals to find and maintain an optimal route (Lihoreau et al., 2012; Reynolds et al., 2013). However, simulations based on bee behaviour and insect neuroanatomy have shown that random excursions and memory for vectors between locations, and then comparisons between total travelled distance, might suffice when visiting the same equally rewarded locations (Le Moël et al., 2019). An animal’s trapline should also change when the environment changes to accommodate increases and decreases in the rewards a location offers. For example, bumblebees trained to forage on five equally rewarding artificial flowers first developed the shortest possible route as a trapline. However, when one of the flowers suddenly contained a higher reward, the bees prioritized visiting this location, even though the new route was now no longer the shortest (Lihoreau et al., 2011). This scenario is relevant to central-place foragers that feed from a resource that is stable in space but that varies with time. Nectar provided by flowers is such a resource, and so we might expect to observe similar development of, and then changes to, traplines in other nectarivores.

Like bumblebees, hummingbirds are a useful system for investigating how different types of information influence foraging decisions and responses to changes in resource distribution. First, flight for these birds is energetically costly, and they need to feed from many flowers in a day (Gass, & Garrison, 1999; Kodric-Brown, & Brown, 1978). As flowers that contain nectar are visually indistinguishable from those that do not (Irwin, 2000), hummingbirds need to remember their locations to avoid visiting flowers they have recently emptied. Second, rufous hummingbirds (*Selasphorus rufus*), at least, are easily trained to feed from artificial flowers and will revisit replenished flowers every 10 minutes or so throughout the day. While tracking wild animals through their territories is still challenging (although new technologies are being developed; e.g., Riotte-Lambert et al., 2017; Rousseu et al., 2014), by training hummingbirds to feed from several locations we can both describe their behaviour and the type of information (such as distance between location, quality of resources) they use when they fly repeated sequences around several rewarding locations. For example, field experiments have shown that these territorial birds primarily use spatial location to relocate rewarding flowers (Hurly, & Healy, 1996; Tello-Ramos et al., 2014), as do food-storing birds like chickadees and tits (Sherry & Hoshooley, 2007; Sherry et al., 1981), using nearby landmarks (Healy, & Hurly, 1998; Pritchard et al., 2015). Furthermore, rufous hummingbirds will repeat the order in which they visit five artificial flowers, develop the shortest possible trapline around all the flowers (Tello-Ramos et al., 2015), and when presented with patches of artificial flowers that refill at regular intervals, they will also incorporate the temporal information into their traplining sequences (Tello-Ramos et al., 2019).

Given that rufous hummingbirds will learn a stable spatiotemporal pattern of food availability, here we tested whether they would change their routes in response to increases or decreases in the value of one of the locations along their trapline. The questions were whether hummingbirds, like the bumblebees, would prioritize a higher reward over a short route and, how decreases in sucrose concentration at different points along the trapline would affect the hummingbirds’ traplines. Thus, we allowed hummingbirds to feed from an array of five artificial flowers until they had developed a repeatable sequence (Lihoreau et al., 2011; Tello-Ramos et al. 2015). Then, in one manipulation, in the flower that the birds were most likely to visit third in their trapline, we increased the sucrose concentration from 25% to 45%, a much-preferred concentration (Morgan et al., 2014; Fig. 1), but maintained the volume constant in all flowers. In two further manipulations, we reduced the sucrose concentration from 25% to 5% in either the flower the birds were most likely to visit first along their trapline, or the flower they were most likely to visit second.

**Fig. 1:**
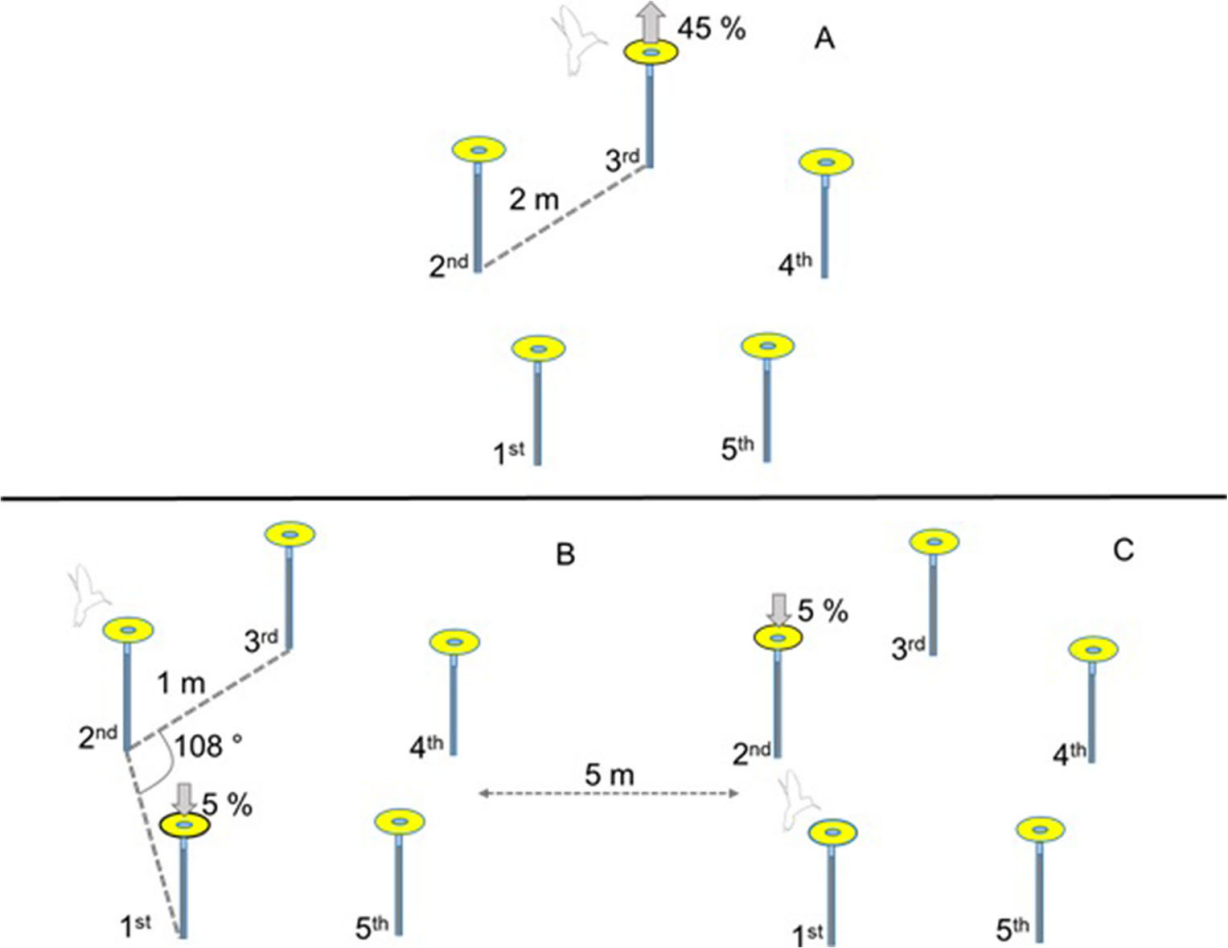
Diagram of the experimental protocol for the second phase of the three experiments. The five artificial flowers were arranged in a pentagon, with flowers 2 m apart for Experiment 1 and 1 m in Experiments 2 and 3. During Phase 1 of each experiment, all flowers were equally rewarded with the same amount of 25% sucrose solution. During Phase 2, however, we increased or lowered the sucrose concentration of one of the flowers. **A** In Experiment 1, we increased the concentration of the flower most likely to be visited in third place during Phase 1. **B** In Experiment 2, we decreased the concentration of the sucrose from 25% to 5% in the flower that was visited most often first during Phase 1. **C** In Experiment 3, we decreased the sucrose concentration of the flower visited most often in second place during Phase 1

Given that the flowers were arranged in a pentagon and that the hummingbirds tend to approach and leave the array from the same direction (from and back to its main perch) the shortest route to visit all flowers would be along the perimeter, with the hummingbird moving in the same clockwise or counterclockwise direction towards the closest flower, without backtracking. If, like other nectarivores, hummingbirds change their traplines to prioritize a high reward while avoiding a low reward, these birds should change the origin of their traplines to visit the now higher rewarding flower first. During the second and third manipulations, although we expected birds to stop visiting the flower containing a low reward, we expected the traplines to change differently depending on where the flower with a lower concentration of sucrose was located within the trapline (i.e., the first or the second flower). Because hummingbirds would have already developed the shortest route around the flower array, if hummingbirds prioritize a short route when we decreased the sucrose solution in the first flower, the birds should change the origin of their traplines and begin their route from the second preferred flower. When we decreased the second flower in the traplines, birds should reverse the direction of their trapline since that would allow them to visit all rewarded flowers with the shortest route.

## Methods

### Subjects and experimental site

We tested a total of 21 free-living territorial male rufous hummingbirds in the Westcastle Valley in south-western Alberta, Canada (N49.349153, W114.410864). First, we placed 26 artificial feeders containing 20% sucrose solution weight by weight (w/w) at sites along the Westcastle Valley. Adult rufous hummingbirds return to this valley from northern Mexico in May for breeding, where males establish a territory around a feeder (Bailey et al. 2013). Once a bird was defending a territory, we caught him and marked him on his breast feathers with coloured nontoxic waterproof ink (Jiffy Eco-marker Ink), and then immediately released him. A day after a bird was marked, we returned to his territory and trained him to feed from an artificial flower (described below).

The University of St Andrews Ethical Committee and the University of Lethbridge Animal Welfare Committee approved all work, which was also conducted under permit from the Alberta Sustainable Resource Development and Environment Canada.

### Experimental procedure

#### Flower training

Artificial flowers consisted of a syringe tip surrounded by a disc (diameter 6 cm) made of yellow foam and attached to a 60-cm wooden stake. The syringe tip contained 100 μl of 25% (w/w) sucrose solution. Once we had trained a hummingbird to feed from an artificial flower, we presented five of these flowers arrayed in a pentagon such that the flowers were 2 m from their nearest neighbours; each flower now contained 15 μl of 25% sucrose (see Fig. 1).

After each foraging bout by the bird to the array, we replaced 15μl in all flowers. If the bird had not finished the sucrose solution in any of the flowers that he had visited, we emptied and refilled those flowers. For each bout we recorded the sequence in which the bird visited the flowers (e.g., 1-2-3-4-5, 1-2-3-5-4, or some other). All visits were included, even those rare occasions (5.6% of all visits) when a bird visited an empty flower (e.g., 1-2-3-4-5-1). Territorial male rufous hummingbirds only fill their crops to a 10%, capacity preferring small but frequent meals as they will benefit from carrying less weight when chasing off an intruder or displaying to a female (Hixon & Carpenter, 1988). Therefore, our hummingbirds sometimes did not visit all five flowers in the array, even when the total amount of sucrose solution in the array was as little as 75 μl. For this reason, and to encourage the hummingbirds to visit all flowers within the array, instead of increasing or decreasing the volume of the reward as it was done for the traplining study on bumblebees (Lihoreau et al., 2011), we manipulated the sugar concentration.

### Experiment 1. Increasing the sucrose concentration of the third flower

Experiment 1 was conducted with nine male rufous hummingbirds (each tested in their own territory) and consisted of two phases. During the first phase, birds were allowed to feed from an array (as described above) of five equally rewarded artificial flowers (15 μl of 25%) for 45 bouts. Across all 45 bouts, we determined which flower each bird had visited third most frequently. When the bird next visited the array during the second phase, the third flower contained 15 μl of 45% sucrose (the other four flowers contained 15 μl of 25%). We allowed the bird to forage from this array for a further 45 bouts and recorded the order of flower visitation (see Fig. 1a). As the hummingbirds visit the array every 10 minutes and we presented the arrays for an average of 9 hours every day, hummingbirds completed the two phases within 2 days.

### Experiment 2. Decreasing the sucrose concentration of the first flower

Experiment 2 was conducted with 12 different male rufous hummingbirds, again on each bird’s own territory. As with Experiment 1, this experiment had two phases. During the first phase a bird was allowed to visit a five-flower array for 45 consecutive bouts. In this pentagonal array, the flowers were 1 m from their nearest neighbour (see Fig. 1b). We changed the distance between flowers to be able to fit two flower arrays within the same region of a bird’s territory. In Phase 1, all the array flowers contained the same amount and concentration of sucrose (15–25 μl of 25% [w/w], depending on the bird). As birds differed during training in the average volume of sucrose they drank, we adjusted the amount of sucrose in the experimental flowers between birds to ensure that they usually visited all flowers in the array with neither early termination of a bout or excessive revisiting of flowers. During Phase 2, we decreased the sucrose concentration in the flower that the bird had most often visited first from 25% to 5% (w/w). We then allowed the bird to visit the array for another 45 bouts and recorded the sequence in which he visited the flowers.

### Experiment 3. Lowering the sucrose concentration of the second flower

Ten of the 12 birds tested in Experiment 2 were also tested in Experiment 3. The order in which birds performed Experiments 2 and 3 was counterbalanced. After the second phase of Experiment 2 or 3 had finished, we moved the five flowers to a new location that was 5 m from that of the previous location. We did this to reduce any impact of the bird’s previous experience of the spatial distribution of flower quality in flower arrays. Again, during Phase 1, the birds visited the five equally rewarded flowers for 45 consecutive bouts. For each bout, we recorded the order in which the bird fed from the flowers. For each bird, we then determined which flower he had visited most often second and then decreased the concentration in that flower from 25% to 5% (see Fig. 1c). During the 45 bouts of Phase 2, we recorded the order in which birds visited the flowers.

#### Statistical analyses

There are 120 different sequences that can be used to visit five locations when each location is visited once, but because the birds had only 45 bouts to visit the flower array during each phase of the experiment, the maximum possible number of different sequences was 45. If a bird repeated a sequence more often than expected by chance, we regarded that sequence as one of the bird’s trapline. Birds flew a median of 24 different sequences. Rather than setting the number of possible routes at 45, by using each bird’s number of individual routes we were more conservative when assessing whether a bird had repeated a trapline more often than chance. For example, if during the 45 bouts a bird flew around the flowers in only 21 different sequences, and repeated one of those sequences 12 times, then he flew that sequence more often than expected by chance (binomial test with an expected proportion of 1 /21, *Z* = 6.9, *p* < .001). Therefore, to determine if a bird had repeated any sequence more times than that expected by chance, we used binomial tests for each of the phases of each of the three experiments. Sequences of all lengths (e.g., 3, 4, or 5 flowers visited) were considered equal and were included in the count of different sequences used by a bird. To compare the number of different sequences used and the number of times the focus flower was visited during Phase 1 and Phase 2 of Experiment 1, we used Wilcoxon matched-pair tests. To compare the number of different sequences used and the number of times the focus flowers were visited during Phase 1 and Phase 2 of Experiments 2 and 3, we used Friedman’s analyses of variance (ANOVAs) for repeated measures.

To examine whether there was a pattern in the way hummingbirds changed their traplines depending on which flower had its sucrose concentration decreased during the second and third experiments, we calculated the probability of different transitions between flowers before and after the sucrose solution manipulation using Markovian chain likelihood ratio tests. Markov chains are stochastic models that compute the probability of a sequence of possible events, which in this case are movements between flowers, based on the history of a finite number of preceding movements (Bakeman & Gottman, 1997; Ivanouw, 2007). For this, observed matrices of transitions between flowers were calculated by counting the number of transitions between all flowers (e.g., number of times a bird visited flower “5” after having visited flower “4”). Then, as an independent model, an expected matrix was calculated using the frequency of transitions between flowers and a simple probability matrix calculated based on the total number of transitions made by each bird. A likelihood ratio test was then used to compare the transition matrix with the expected matrix. For example, if a bird visited flower “5” after having visited flower “4” 10/45 times, then the final calculated *Z*-score in the matrix for that transition would be >1.96, which meant that the transition “5–4” was more likely than expected by chance at *p* < .05 (see Supplementary material 1). We used binomial tests to determine whether birds changed the origin and the direction of their traplines more often than expected by chance. In order to compare the number of times focus flowers were visited in first and second place, we used Wilcoxon signed-rank tests. All analyses were conducted using R (Version. 4.0.4; R Core Team, 2021).

## Results

As expected, throughout all three experiments and through the two different phases, hummingbirds developed one or two traplines that were repeated more often than expected by chance (see Tables 1 and 2). Those traplines changed in different ways in response to the different sucrose manipulations.

**Table 1:** Trapline repeated most times by each bird during Experiments 1 in Phase 1 and 2 of each experiment (E1P1, E1P2)

Bird	E1P1	*p* value	E1P2	*p* value
1	1-2-3 (6)	.002	2-3-4 (7)	.001
2	1-2-3 (6)	.019	1-5-4 (7)	.005
3	5-4-3-2-1 (4)	.16*	1-5-4 (11)	.000
4	1-5-3 (3)	.12*	1-2-3 (4)	.05
5	5-1-2 (3)	.21*	5-1-2 (6)	.007
6	5-4-3 (7)	.003	5-4-3 (9)	.003
7	1-2-3-4-5 (5)	.03	1-5-4-3-2 (7)	.005
8	3-4-5-1-2 (7)	.002	2-1-5 (7)	.005
9	4-5 (4)	.062*	1-2-3 (6)	.007

*Note.* The preferred sequence is listed by the arbitrary numbers designated a priori by the experimenter where “1–2” indicates the bird visiting Flower 1 followed by Flower 2. The number of times that exact sequence was repeated is in the brackets next to the sequence. The asterisk indicates the non-significant result (*p* > .05) and 0.00 indicates *p* < .01 calculated by binomial tests.

**Table 2:** Trapline repeated most times by each bird during Experiments 2 and 3 in Phases 1 and 2 of each experiment (E2P1, E2P2, E3P1 and E3P2)

Bird	E2P1	*p* value	E2P2	*p* value	E3P1	*p* value	E3P2	*p* value
1	5-1-2-3 (6)	.01	1-2-3-4 (9)	.00				
2	5-1-2 (8)	.00	1-2-3 (10)	.00	5-4-3-2 (5)	.01	5-4-3-2 (13)	.00
3	5-4-3 (8)	.00	4-2-1 (8)	.00	1-5-4-3-2 (8)	.00	1-2-3-4 (6)	.01
4	1-5-4-3-2 (6)	.00	5-4-3-2 (13)	.00	2-3-4-5-1 (7)	.00	3-4-5-1 (8)	.00
5	1-5-4-3 (3)	.19*	1-2 (6)	.01				
6	3-4-5-1 (5)	.04	2-1-5-4 (9)	.00	2-1-5-4 (5)	.01	5-4 (5)	.02
7	2-1-5-4 (6)	.02	1-5-4-3 (17)	.00	1-2-3-4 (5)	.02	5-4-3-2 (6)	.01
8	1-5-4-3-2 (17)	.00	5-4-3-2 (19)	.00	3-2-1-5-4 (14)	.00	3-4-5-1 (27)	.00
9	2-1-5-4 (5)	.02	3-4-5-1 (11)	.00	3-4-5-1-2 (11)	.00	3-2-1-5 (14)	.00
10	2-1-5-4-3 (12)	.00	3-4-5-1 (14)	.00	3-4-5-1-2 (9)	.01	3-4-5-1 (13)	.00
11	2-1-5-4-3 (8)	.00	3-4-5-1 (11)	.00	5-1-2-3-4 (6)	.01	4-5-1-2 (9)	.00
12	1-5-4-3 (9)	.00	5-4-3-2 (17)	.00	1-2-3-4-5 (8)	.00	1-2-3-4 (6)	.01

*Note.* The preferred sequence is listed by the arbitrary numbers designated a priori by the experimenter where “1–2” indicates the bird visiting Flower 1 followed by Flower 2. The number of times that exact sequence was repeated is in the brackets next to the sequence. The asterisk indicates the non-significant result (*p* > .05) and 0.00 indicates *p* < .01 calculated by binomial tests.

### Experiment 1. Increased sucrose concentration in the third flower

**Fig. 2:**
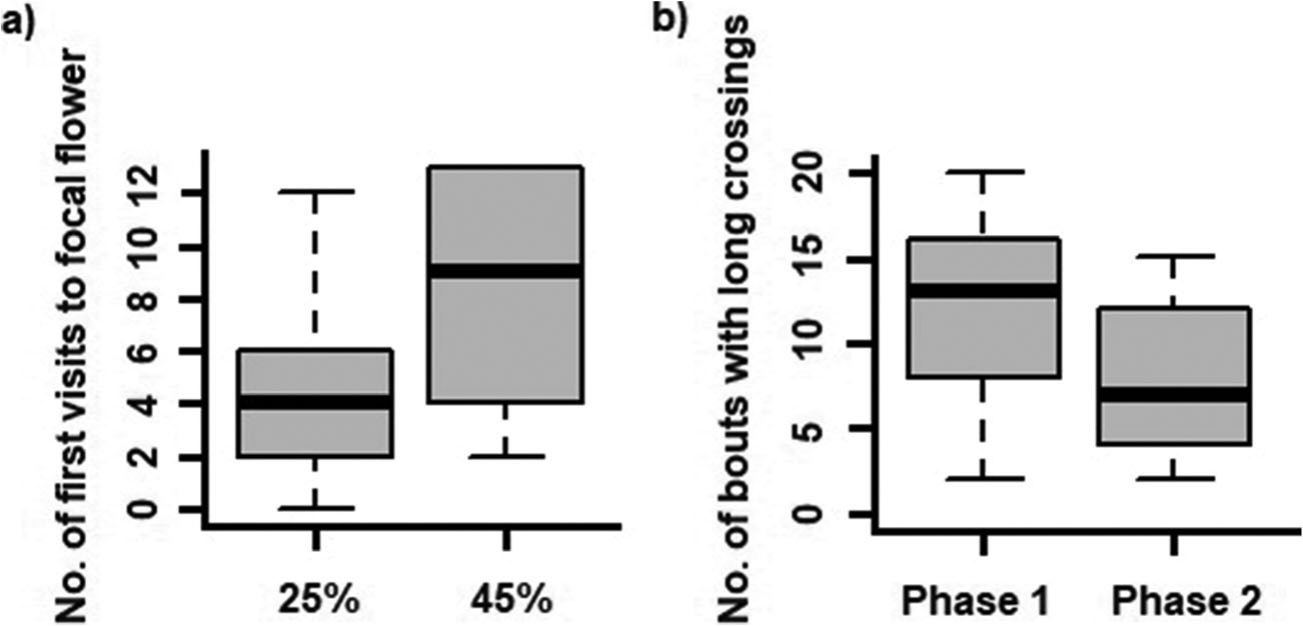
**a** Median percentage of bouts where birds visited the focal flower third during Phase 1 and in Phase 2 when this flower contained 45% sucrose solution. The boxes show the median and the first and third quartiles, and the bars represent the minimum and maximum values (*N* = 9). **b** Median percentage of bouts where birds visited flowers that were not contiguous and therefore included diagonal flights across the array during Phase 1 and Phase 2 when the sucrose concentration in the third flower had been increased to 45%. The boxes show the median and the first and third quartiles (*N* = 9)

In Phase 1, five of the nine birds developed at least one repeatable trapline (binomial tests, *p* < .05; see Table 1). In Phase 2, when we had increased the concentration of the sucrose solution in the flower that each bird had been most likely to visit third in Phase 1 from 25% to 45%, the birds flew significantly fewer different sequences (No. of sequences used in Phase 1: median = 24, IQR = 23–30, compared with no. of sequences used in Phase 2: median = 22, IQR = 21–26, Wilcoxon matched-pairs test *W* = 36, *p* = .014, *r* = −.81, *N* = 9). Eight of the nine birds used at least one trapline more often than expected by chance (binomial tests, *p* < 0.05) and the remaining bird’s use of one route was nearly significant (*p* = .051). In Phase 2 hummingbirds visited the flower containing the 45% sucrose solution significantly more often than they had in Phase 1 when that flower had contained a reward equal to that of the other flowers (No. of visits in Phase 1: median = 32, IQR = 30–33, compared with no. of visits in Phase 2: median = 39, IQR = 35–40; Wilcoxon matched-pairs test *W* = 1, *p* = .02, *r* = −.77, *N* = 9). The birds also increased the number of times they visited the highly rewarded flower *first*, but this change was not significant (No. of visits to the ‘third’ flower first in Phase 1: median = 4, IQR = 2–6, compared with no. of visits to the ‘third’ flower first in Phase 2: median = 9, IQR = 4–13; Wilcoxon matched-pairs test *W* = 8.5, *p* = .10, *r* = −.53, *N* = 9; see Fig. 2a).

During Phase 1, just over a quarter (median = 13, IQR = 8–16) of the hummingbirds’ sequences were longer than were optimal because the birds flew routes without visiting all contiguous flowers one after the other (e.g., instead of visiting flowers 1-2-3-4-5, the bird visited flowers 1-2-4-5-3). When we increased the concentration of sucrose in the ‘third’ flower, the hummingbirds flew significantly fewer of these longer sequences (median = 7, IQR = 4–12; Wilcoxon matched-pairs test *W* = 33, *p* = .042 *r* = −.67, *N* = 9; see Fig. 2b). This change significantly decreased the distance of the sequences flown by birds (distance of sequences flown in Phase 1: median = 4.9 m, IQR = 4.26–6.20, compared with the distance of sequences flown in Phase 2: median = 3.7 m , IQR = 4.3–5.5; Wilcoxon matched-pairs test *W* = 42, *p* = .01 *r* = −.77, *N* = 9). Long sequences were interspersed equally throughout both phases and during the second phase. Birds did not change, however, the number of times they visited all five flowers within the array (Phase 1: median = 8.88, IQR = 1–17, compared with Phase 2: median = 6.22 , IQR = 1–12; Wilcoxon matched-pairs test *W* = 26, *p* = .29, *r* = −.35, *N* = 9) or indeed the mean number of flowers visited on each bout (No. flowers visited in Phase 1: median = 3.22, IQR = 2.95–4, compared with no. of flowers visited in Phase 2: median = 3.04 , IQR = 2.73–3.71;Wilcoxon matched-pairs test *W* = 37, *p* = .09, *r* = −.55, *N* = 9).

### Experiments 2 and 3. Decreased sucrose concentration in the first and second flowers

Ten of the 12 birds completed both Experiments 2 and 3. During both phases in both Experiments 2 and 3, all birds repeated at least one sequence more often than expected by chance (*p* < .05; see Table 2). There was no difference in the number of different sequences used by the birds across Phases 1 and 2 of either Experiment 2 or 3 (No. of different sequences used during Experiment 2, Phase1: median = 26, IQR = 23.58–28.25; Phase 2: median = 22.5, IQR = 18.75–25; Experiment 3, Phase 1: median = 25, IQR = 23–27.75; Phase 2: median = 22.5, IQR = 17.75–24.5); Friedman’s ANOVA, χ^2^(3) = 6.1795, *p* = .103, *N* = 10.

**Fig. 3:**
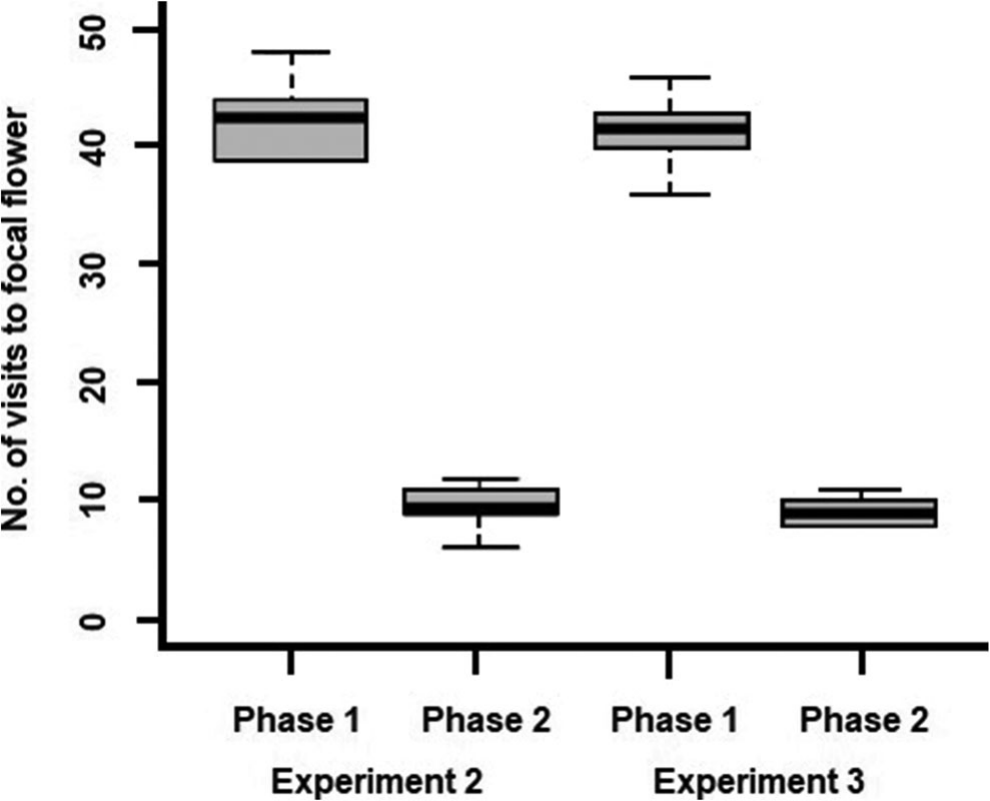
Median number of visits to the focal flower (flower that during Phase 2 contained 5% sucrose solution) during Experiments 2 and 3. The boxes show the median and the first and third quartile (*N* = 12)

When a flower contained 5% sucrose solution, the number of times birds visited this flower decreased significantly between Phase 1 and Phase 2, Friedman’s ANOVA, χ^2^(3) = 24.4, *p* < .001, *n* = 10. This effect was independent of experiment type (first or second flower modified) to such a degree that the results of the two experiments are almost identical (see Fig. 3). Post hoc tests with Bonferroni corrections applied showed that birds visited the focus flower in Phase 2 significantly less often than they had visited it in Phase 1 in both Experiments 2 and 3 (*difference* = 20 for both cases). In all cases, the critical difference (α = 0.05 corrected for the number of tests) was 15.23 (see Fig. 3).

**Fig. 4:**
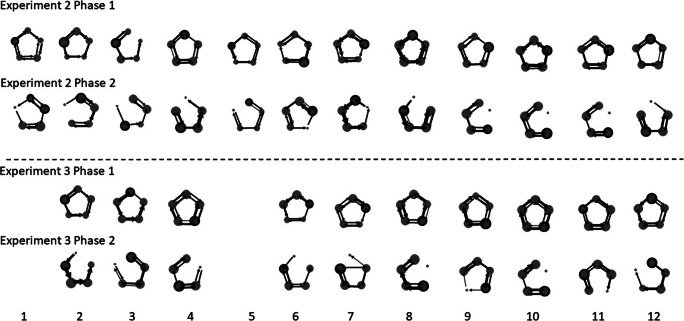
Markovian chain transition diagrams for each bird (numbers from 1 to 12) for each phase of each experiments 2 and 3 (e.g., Experiment 2, Phase 1). The arrows represent the transitions that the bird made significantly more frequently than at chance (*p* < .05, where the *Z* score >1.96). The arrows are also proportional to the size of the score: the wider the arrow, the larger the score. The circle size is proportional to the frequency of visits it represents. Circles with black outlines represent the flowers that were visited more often in first place

The Markovian chain diagrams for each bird (see Fig. 4) show the frequency with which each flower was visited (the size of the circle is proportional to the visitation frequency and the circles with black outlines represent the flowers that were visited first more times) and the transitions that were made more often than expected by chance (the width of the arrow is proportional to the likelihood of the occurrence of that transition). All of the birds made significant transitions in every experimental phase. Remarkably, only one bird (Bird 7) flew between nonadjacent flowers more often than predicted by chance (17 times from Flower 5 to Flower 2), and this occurred in only one phase of the experiment (E3P2). All other birds’ sequences included significant transitions only between adjacent flowers (see Fig. 4; Supplementary material1).

When the sucrose concentration in the *first* flower of a bird’s trapline was decreased, the birds stopped visiting that flower and most frequently changed the origin of the trapline by visiting the previous second flower first. Ten of the 12 birds changed the origin of their bouts by visiting first the flower that had been most often visited in second place in the previous phase (binomial test where the probability of first visiting one of the 25% sucrose flowers was equal to 1/4, 264/540, *Z* = 12.82, *p* < .001). Indeed, the number of times the original first flower had been visited first in Phase 1 and the number of times the “new” first flower was visited first in Phase 2 did not differ (Experiment 2 Phase1: median = 23.50, IQR = 19.5–29.5; Experiment 2 Phase 2: median = 23, IQR = 21.5–28; Wilcoxon signed-rank test, *W* = 36.5, *p* = .78, *r* = −.07, *N* =12).

When we lowered the concentration of sucrose in the *second* flower in Phase 2, however, 7 out of 10 birds continued to visit first the flower they had visited first in Phase 1: there was no significant difference in the number of times the birds visited the first flower during Phases 1 and 2 (No. of times the first flower was visited in first place in Experiment 3, Phase1: median = 18, IQR = 17–23; Phase 2: median = 19.5, IQR = 15–26.75; Wilcoxon signed-rank test in *W* = 22.5, *p* > .99, *r* < .001, *N* = 10). During Phase 2, seven out of 10 hummingbirds kept the origin of their traplines but reversed the direction of the rest of the visits within a bout: after visiting the previous first flower, birds visited the flower that was to the opposite side as the previous second flower (binomial test where the probability of visiting one of the 25% sucrose in second place flowers was equal to 1/3, 186/450, *Z* = 3.6, *p* < .001; see Fig. 4). For example, if during the first phase a bird had visited the flowers in the sequence: 1-2-3-4-5 most often, when the sucrose in Flower 2 was decreased, the bird was more likely to visit the flowers in a 1-5-4-3 sequence than in a 1-3-4-5 sequence (see Fig. 4). There was no difference between the number of times the second flower was visited in second place during Phase 1 and number of times the “new” second flower was visited in second place during Phase 2 (No. of times second flower was visited in second place in Experiment 3, Phase1: median = 17, IQR = 15.25–17.75; Phase 2: median = 18, IQR = 17.25–21.25; Wilcoxon signed-rank test in *W* = 16, *p* > .25, *r* = −.35, *N* =10). There was no pattern in the changes the other three birds made when they visited the array once the concentration of the second flower was lowered. Bird 4 changed the origin and the direction, Bird 6 changed origin but kept the direction and Bird 7 changed origin and incorporated a long crossing (see Fig. 4).

## Discussion

Hummingbirds foraging from an array of five equally rewarded flowers used one or two traplines during most of their foraging bouts. Those traplines were always the shortest possible routes as birds did not include “long” crossings between flowers (i.e., diagonal between two vertices). When the sucrose concentration *increased* in the third flower within a trapline, birds did not change their traplines. Conversely, however, when the sucrose concentration of one of the flowers in their trapline *decreased*, the hummingbirds *did* change their traplines. How they changed their trapline depended on where in the trapline the decrease in reward occurred: when the decrease occurred in the flower that was typically visited first in the trapline the birds changed the trapline origin to the flower they had visited most in second place during the previous phase. However, when the decrease occurred in the flower the bird had most typically visited *second* in the first phase, in the second phase the hummingbirds kept the origin of their traplines, but reversed the direction in which they visited the remaining flowers. Birds did not fly over the poorly rewarded flowers; instead, they modified their traplines in order to continue to use the shortest routes that connected all rewarding flowers.

In Experiment 1, hummingbirds did not prioritize the increased reward. This might seem unexpected because hummingbirds can not only discriminate but also prefer higher concentrations over lower concentrations of sucrose (to about 50% sucrose: Blem et al., 2000). Although our hummingbirds may appear to have behaved differently to bumblebees that experienced an increase in a rewarding location, the bumblebees changed their trapline to begin at the flower containing the increased reward only when the travel route increased by a small amount (18%). When the increased reward was placed in a flower that meant that visiting that flower first would increase the flying route by 42%, bumblebees did not alter their original optimal routes (Lihoreau et al., 2011). In the case of our hummingbirds, visiting the third flower first and then resuming the original trapline would have increased any optimal trapline by 32%. We however, did not observe hummingbirds modifying their traplines in such away. Male rufous hummingbirds exclude competitors from their territories, unlike bees and bumblebees that feed from flowers that are visited by many other pollinators and therefore there might be less pressure on hummingbirds to prioritize visiting the most rewarding flower first. The birds did, however, include the high reward flower in significantly more bouts than during Phase 1, and they did so by also decreasing the mean distance flown between the flowers visited. Going ‘out of their way’ may, however, occur in specific circumstances. Sometimes this is only apparent: for example, wild chacma baboons (*Papio ursinus*) appeared to prioritize certain resources (fruit over seeds; Noser & Byrne, 2007) when they bypassed near resources for rewarding locations further away, but in fact, the baboons travelled first to the far rewarding locations before returning to the near rewarding locations situated near to their sleeping site. But we might expect that animals, including both hummingbirds and bees, will make exceptions when presented with highly rewarding limited resources like pollen for a bee or a possible mate for a hummingbird (Hurly, 2003).

In Experiments 2 and 3, similar to the result from Experiment 1, following the change in reward, birds prioritized those routes that were the shortest, either beginning a shortened trapline at Flower 2, or reversing the direction of their trapline. Perhaps because bumblebees are more stereotyped in the direction they fly, this simple solution was not observed in a similar experimental task (Lihoreau et al., 2011). These authors argued that a change in direction would have required the bees to re-learn all the distances and directions between individual flowers. Because we do not yet know what information the hummingbirds learn when they develop their trapline, we cannot be certain whether they specifically reversed retained information or simply relearned a new trapline. Because 7 out 10 birds in the second phase of Experiment 3, when we lowered the concentration of the second flower, kept the origin of their trapline but reversed the direction in which they flew around the flowers, it seems plausible that these birds did just invert the direction. We do know, however, that wild hummingbirds learn the locations of flowers in relation to visual landmark and that movement vectors are thought to be used over long distances during migration (Pritchard & Healy, 2018). What information they use when moving between multiple locations is yet to be determined, but comparing the traplining behaviour of different taxa might be able to tell us something more about the type of information animals use when solving these multi-location problems.

Hummingbirds changed their traplines to avoid a poor reward, but not to prioritize a rich one. This difference could be explained by hummingbirds valuing these changes in sucrose concentration nonsymmetrically (Hurly, 2003). For example, animals that are on a positive energy trajectory, such as hummingbirds, tend to be risk-averse, since the consequences of a foraging loss are larger than the consequences of an equivalent gain. Furthermore, even though energetically the difference fom an increase of 25% to a 45% sucrose and a decrease from 25% to 5% are equivalent, due to Weber’s law hummingbirds might perceive the decrease as a larger difference and thus be more likely to alter their traplines (Kacelnik & El Mouden 2013).

The comparison of these two unrelated groups appears to provide both general and specific components of traplining (Pritchard et al., 2017; Sherry & Strang, 2015). For example, traplining hummingbirds and bees both prioritize short distances and are not faithful to their traplines (Kembro et al., 2019; Woodgate et al., 2017). Hummingbirds, too, use their preferred traplines interspaced with other “sampling” sequences and frequently visit just a subset of the flowers along the preferred trapline (Tello-Ramos et al., 2015). However, while individual bees have a strong preference for the direction in which they fly their trapline (either clockwise or anticlockwise; Lihoreau et al., 2011), hummingbirds do not. It seems, then, that hummingbird traplines are more flexible and variable than those reported for bees. This difference in foraging behaviour might be due to the different ecologies of hummingbirds and bees: while a territorial rufous hummingbird is vigilant for both competitors and potential mates, a bumblebee is one member of a colony specialized in foraging. A bumblebee will be collecting nectar and pollen not just for its individual consumption but for the needs of the hive, and thus bumblebees in general might be more sensitive to changes in the quality of the resource or more willing to accept personal costs. Our hummingbirds need to worry only about their individual fitness and thus they can decide whether to prioritize a short trapline over a high reward or prioritize avoiding a poor reward over a short trapline. There is another difference between hummingbirds and bumblebees. While territorial males rufus hummingbirds rarely fill their crops (they only drink about 10% of their crop capacity), bumblebees will visit as many flowers as possible to fill their stomach before returning home. Given this difference, we manipulated the concentration of the sucrose reward rather than the volume and thus the two studies should be compared with caution. Whether or not the similarities in foraging coincide with homologous information use is not yet clear (Boisvert, & Sherry, 2006; Boisvert et al., 2007; Pritchard et al., 2017; Tello-Ramos et al., 2019).

The problem of multi-location travel is common to animals searching for different types of resources (i.e., food, mates, and shelter) and is even relevant to “our own traplining behaviours” (e.g., food-supply chains that can easily be interrupted if one step of the chain is compromised). Understanding the behavioural “algorithm” that animals use to optimize their routes, or the minimum amount of information needed to choose optimal routes under different circumstances may, in turn, help us to reduce the footprint of the things we buy, how we travel, or to better prepare for unforeseen interruptions in supply chains.

## Supplementary Information

ESM 1 (DOCX 59 kb)
