# Supplementary material for: Hummingbirds modify their routes to avoid a poor location
Source: Learn Behav. 2021 Aug 2;50(1):89–98. doi: 10.3758/s13420-021-00476-3 (PMC8979907; doi:10.3758/s13420-021-00476-3)
Supplement: Supplementary file 1 — (DOCX 59 kb) [file 13420_2021_476_MOESM1_ESM.docx]

Supplementary material 1. Table with the G^2^ statistics and p-values from Markovian Chain Likelihood ratio test comparing observed and expected matrices. Transitions that had a Z-score > 1.96 were made more often than chance at a significant level of 0.05 and are in bold. Z- score for each flower F1-F5.

| **Bird** | **Ex/Phase** | **G^2^** | **P value** | **Z score for each transition** | | | | | |
| --- | --- | --- | --- | --- | --- | --- | --- | --- | --- |
| **1** | E2P1 | 119.30 | <0 .0001 |  | **F1** | **F2** | **F3** | **F4** | **F5** |
|  |  |  |  | **F1** | -2.37 | **4.78** | -2.66 | -1.29 | 1.73 |
|  |  |  |  | **F2** | -0.17 | -2.28 | **4.69** | -1.34 | -1.48 |
|  |  |  |  | **F3** | -1.70 | **2.63** | -2.33 | **2.65** | -1.27 |
|  |  |  |  | **F4** | -2.30 | -2.17 | **3.72** | -2.30 | **3.06** |
|  |  |  |  | **F5** | **5.94** | -2.67 | -3.11 | **2.37** | -2.17 |
|  | E2P2 | 103.34 | < 0.0001 |  | **F1** | **F2** | **F3** | **F4** | **F5** |
|  |  |  |  | **F1** | -1.90 | **2.93** | -0.57 | -2.10 | **2.42** |
|  |  |  |  | **F2** | **5.0** | -3.33 | **2.72** | -3.14 | -1.41 |
|  |  |  |  | **F3** | -2.26 | 1.66 | -4.18 | **5.69** | -1.45 |
|  |  |  |  | **F4** | -1.31 | -0.27 | **3.34** | -2.61 | 0.79 |
|  |  |  |  | **F5** | 0.12 | -1.08 | -1.50 | **2.44** | 0.12 |
| **2** | E2P1 | 124.78 | < 0.0001 |  | **F1** | **F2** | **F3** | **F4** | **F5** |
|  |  |  |  | **F1** | -3.29 | **5.85** | -3.11 | -2.73 | **3.38** |
|  |  |  |  | **F2** | 0.19 | -2.15 | **5.07** | -2.02 | -1.05 |
|  |  |  |  | **F3** | 0.11 | -0.57 | -1.62 | 1.46 | 0.66 |
|  |  |  |  | **F4** | -1.65 | -1.70 | **4.98** | -1.59 | 0.10 |
|  |  |  |  | **F5** | **4.13** | -2.32 | -3.39 | **4.33** | -3.0 |
|  | E2P2 | 113.10 | < 0.0001 |  | **F1** | **F2** | **F3** | **F4** | **F5** |
|  |  |  |  | **F1** | -2.70 | **6.64** | -4.09 | -1.29 | 1.39 |
|  |  |  |  | **F2** | **2.88** | -4.06 | **5.47** | -2.80 | -1.70 |
|  |  |  |  | **F3** | -1.41 | -0.39 | -2.64 | **4.93** | -0.79 |
|  |  |  |  | **F4** | -0.79 | -1.71 | **2.61** | -1.50 | 1.72 |
|  |  |  |  | **F5** | **2.26** | -1.82 | -1.31 | 1.64 | -0.33 |
|  | E3P1 | 143.00 | < 0.0001 |  | **F1** | **F2** | **F3** | **F4** | **F5** |
|  |  |  |  | **F1** | -2.53 | **4.13** | -3.16 | -2.36 | **3.84** |
|  |  |  |  | **F2** | 0.56 | -2.57 | **4.63** | -2.08 | -0.54 |
|  |  |  |  | **F3** | -1.88 | **3.42** | -2.41 | 1.71 | -1.22 |
|  |  |  |  | **F4** | -1.88 | -2.15 | **5.49** | -2.15 | 0.56 |
|  |  |  |  | **F5** | **5.18** | -2.90 | -3.45 | **4.40** | -2.69 |
|  | E3P2 | 182.27 | < 0.0001 |  | **F1** | **F2** | **F3** | **F4** | **F5** |
|  |  |  |  | **F1** | 0.43 | 0.30 | -2.11 | -2.16 | **5.58** |
|  |  |  |  | **F2** | 1.34 | -1.51 | 1.19 | -1.56 | 1.49 |
|  |  |  |  | **F3** | -0.50 | **7.77** | -3.98 | -1.07 | -1.70 |
|  |  |  |  | **F4** | -0.87 | -3.19 | **8.80** | -4.98 | -1.08 |
|  |  |  |  | **F5** | 0.07 | -3.06 | -4.72 | **8.63** | -2.12 |
| **3** | E2P1 | 77.72 | < 0.0001 |  | **F1** | **F2** | **F3** | **F4** | **F5** |
|  |  |  |  | **F1** | -2.14 | 1.62 | -0.71 | -0.15 | **2.24** |
|  |  |  |  | **F2** | 0.98 | -1.12 | 1.34 | -0.93 | -0.50 |
|  |  |  |  | **F3** | -1.29 | **3.40** | -2.07 | 0.86 | -0.71 |
|  |  |  |  | **F4** | -0.03 | -1.51 | **5.02** | -4.10 | 0.90 |
|  |  |  |  | **F5** | **2.07** | -1.65 | -3.74 | **4.16** | -1.80 |
|  | E2P2 | 76.95 | < 0.0001 |  | **F1** | **F2** | **F3** | **F4** | **F5** |
|  |  |  |  | **F1** | -2.20 | **3.08** | -1.63 | 0.66 | -0.02 |
|  |  |  |  | **F2** | **4.39** | -4.31 | 1.78 | -2.03 | -0.07 |
|  |  |  |  | **F3** | -1.86 | **3.19** | -2.53 | 1.56 | -0.60 |
|  |  |  |  | **F4** | -1.40 | 0.47 | **2.62** | -2.31 | 0.49 |
|  |  |  |  | **F5** | 0.64 | -2.11 | -1.34 | **3.58** | 0.20 |
|  | E3P1 | 162.40 | < 0.0001 |  | **F1** | **F2** | **F3** | **F4** | **F5** |
|  |  |  |  | **F1** | -2.02 | 1.29 | -2.39 | -2.10 | **5.57** |
|  |  |  |  | **F2** | **4.05** | -2.29 | 1.39 | -1.58 | -0.94 |
|  |  |  |  | **F3** | -2.02 | **7.17** | -3.29 | -0.24 | -2.33 |
|  |  |  |  | **F4** | -1.46 | -3.22 | **7.01** | -3.02 | 0.30 |
|  |  |  |  | **F5** | 2.01 | -3.15 | -2.38 | **6.37** | -2.62 |
|  | E3P2 | 108.46 | < 0.0001 |  | **F1** | **F2** | **F3** | **F4** | **F5** |
|  |  |  |  | **F1** | -2.78 | **2.97** | -1.84 | 1.36 | 0.16 |
|  |  |  |  | **F2** | **5.32** | -3.98 | 1.87 | -2.35 | -0.79 |
|  |  |  |  | **F3** | -2.22 | **4.44** | -3.51 | **1.96** | -1.21 |
|  |  |  |  | **F4** | -1.33 | -2.64 | **4.86** | -2.51 | **2.07** |
|  |  |  |  | **F5** | 0.94 | -1.07 | -1.71 | **2.18** | -0.10 |
| **4** | E2P1 | 147.77 | < 0.0001 |  | **F1** | **F2** | **F3** | **F4** | **F5** |
|  |  |  |  | **F1** | -2.47 | **3.59** | -2.06 | -2.86 | **3.34** |
|  |  |  |  | **F2** | 1.53 | -2.26 | **3.51** | -2.20 | -0.51 |
|  |  |  |  | **F3** | -0.96 | **2.75** | -3.17 | **3.82** | -2.28 |
|  |  |  |  | **F4** | -2.27 | -2.67 | **5.14** | -3.05 | **2.13** |
|  |  |  |  | **F5** | **4.67** | -1.85 | -3.11 | **4.44** | -3.11 |
|  | E2P2 | 141.30 | < 0.0001 |  | **F1** | **F2** | **F3** | **F4** | **F5** |
|  |  |  |  | **F1** | 0.29 | -0.22 | -0.37 | -0.83 | 1.31 |
|  |  |  |  | **F2** | **3.97** | -2.24 | -0.19 | -1.38 | 1.64 |
|  |  |  |  | **F3** | -1.26 | **8.78** | -4.61 | -1.59 | -2.35 |
|  |  |  |  | **F4** | -1.37 | -4.22 | **4.71** | -3.19 | **3.24** |
|  |  |  |  | **F5** | -0.35 | -2.82 | 0.16 | **6.53** | -3.01 |
|  | E3P1 | 198.20 | < 0.0001 |  | **F1** | **F2** | **F3** | **F4** | **F5** |
|  |  |  |  | **F1** | -2.62 | **2.71** | -2.06 | -1.99 | **3.77** |
|  |  |  |  | **F2** | **3.63** | -3.09 | **5.13** | -2.14 | -3.13 |
|  |  |  |  | **F3** | -3.15 | **5.29** | -3.08 | **4.55** | -3.62 |
|  |  |  |  | **F4** | -2.74 | -2.74 | **2.12** | -3.04 | **6.15** |
|  |  |  |  | **F5** | **5.11** | -2.43 | -2.37 | **2.55** | -2.82 |
|  | E3P2 | 119.74 | < 0.0001 |  | **F1** | **F2** | **F3** | **F4** | **F5** |
|  |  |  |  | **F1** | -2.13 | 0.09 | -0.26 | -1.38 | **3.34** |
|  |  |  |  | **F2** | -0.39 | 0.02 | **2.89** | -1.34 | -1.07 |
|  |  |  |  | **F3** | -1.52 | **2.98** | -3.23 | **4.31** | **-1.44** |
|  |  |  |  | **F4** | -2.63 | -1.63 | **4.16** | -3.93 | **3.10** |
|  |  |  |  | **F5** | **6.37** | -1.31 | -2.85 | 1.95 | -4.02 |
| **5** | E2P1 | 100.97 | < 0.0001 |  | **F1** | **F2** | **F3** | **F4** | **F5** |
|  |  |  |  | **F1** | -2.46 | **3.17** | -2.73 | -2.55 | **4.64** |
|  |  |  |  | **F2** | **2.81** | -1.81 | **2.38** | -1.74 | -1.66 |
|  |  |  |  | **F3** | -0.15 | **2.90** | -1.70 | 0.41 | -1.48 |
|  |  |  |  | **F4** | -0.90 | -1.67 | **4.36** | -2.16 | 0.26 |
|  |  |  |  | **F5** | 1.05 | -2.48 | -2.04 | **5.86** | -2.30 |
|  | E2P2 | 69.12 | < 0.0001 |  | **F1** | **F2** | **F3** | **F4** | **F5** |
|  |  |  |  | **F1** | -2.08 | **3.68** | -1.80 | -1.23 | 1.43 |
|  |  |  |  | **F2** | **2.26** | -1.99 | **3.03** | -1.91 | -1.38 |
|  |  |  |  | **F3** | -1.65 | **2.26** | -1.91 | **2.41** | -1.38 |
|  |  |  |  | **F4** | 0.55 | -2.28 | 1.90 | -2.19 | **2.34** |
|  |  |  |  | **F5** | 1.09 | -1.90 | -1.18 | **3.23** | -1.30 |
| **6** | E2P1 | 136.68 | < 0.0001 |  | **F1** | **F2** | **F3** | **F4** | **F5** |
|  |  |  |  | **F1** | -2.29 | 0.59 | -1.77 | -1.18 | **4.56** |
|  |  |  |  | **F2** | **4.31** | -3.21 | 1.30 | -1.36 | -1.38 |
|  |  |  |  | **F3** | -2.87 | **6.63** | -3.34 | **2.90** | -3.07 |
|  |  |  |  | **F4** | -2.66 | -2.99 | **5.25** | -1.78 | **2.39** |
|  |  |  |  | **F5** | **4.14** | -2.00 | -1.61 | 1.16 | -1.73 |
|  | E2P2 | 122.96 | < 0.0001 |  | **F1** | **F2** | **F3** | **F4** | **F5** |
|  |  |  |  | **F1** | -4.44 | **2.19** | -0.40 | -1.89 | **5.06** |
|  |  |  |  | **F2** | **6.80** | -2.11 | -0.54 | -2.58 | -2.89 |
|  |  |  |  | **F3** | -1.98 | **3.15** | 0.12 | 1.51 | -1.66 |
|  |  |  |  | **F4** | -2.40 | -0.32 | **2.39** | -2.05 | **3.30** |
|  |  |  |  | **F5** | 0.71 | -1.85 | -1.07 | **5.30** | -3.73 |
|  | E3P1 | 124.50 | < 0.0001 |  | **F1** | **F2** | **F3** | **F4** | **F5** |
|  |  |  |  | **F1** | -3.89 | 0.64 | -1.77 | -1.25 | **5.79** |
|  |  |  |  | **F2** | **6.40** | -2.18 | 1.47 | -3.06 | -2.78 |
|  |  |  |  | **F3** | -2.29 | **3.41** | -1.18 | **3.14** | -2.46 |
|  |  |  |  | **F4** | -1.94 | -0.15 | **2.76** | -2.17 | 1.95 |
|  |  |  |  | **F5** | 1.34 | -1.24 | -1.05 | **3.91** | -3.16 |
|  | E3P2 | 106.05 | < 0.0001 |  | **F1** | **F2** | **F3** | **F4** | **F5** |
|  |  |  |  | **F1** | -0.20 | 0.44 | -1.67 | -1.87 | **3.66** |
|  |  |  |  | **F2** | 1.78 | -2.26 | **4.84** | -2.54 | -1.54 |
|  |  |  |  | **F3** | -1.25 | **2.94** | -3.32 | **3.50** | -2.21 |
|  |  |  |  | **F4** | -1.34 | -0.95 | **2.37** | -3.80 | **3.54** |
|  |  |  |  | **F5** | 1.05 | -0.07 | -2.6 | **4.29** | -2.57 |
| **7** | E2P1 | 165.78 | < 0.0001 |  | **F1** | **F2** | **F3** | **F4** | **F5** |
|  |  |  |  | **F1** | -2.89 | **2.72** | -2.89 | -3.43 | **6.43** |
|  |  |  |  | **F2** | **5.63** | -2.01 | **3.72** | -3.50 | -3.45 |
|  |  |  |  | **F3** | -1.27 | 1.01 | -1.86 | **4.79** | -2.54 |
|  |  |  |  | **F4** | -2.29 | -0.19 | **3.03** | -2.75 | **2.12** |
|  |  |  |  | **F5** | 0.37 | -1.48 | -2.29 | **6.11** | -3.07 |
|  | E2P2 | 184.16 | < 0.0001 |  | **F1** | **F2** | **F3** | **F4** | **F5** |
|  |  |  |  | **F1** | -2.01 | -0.98 | -2.48 | -4.77 | **8.93** |
|  |  |  |  | **F2** | **5.16** | -0.14 | 0.24 | -2.01 | -1.46 |
|  |  |  |  | **F3** | -0.31 | **3.10** | -1.55 | 1.80 | -1.93 |
|  |  |  |  | **F4** | -1.61 | 0.13 | **7.51** | -4.04 | -2.05 |
|  |  |  |  | **F5** | 0.37 | -0.98 | -3.71 | **8.71** | -4.85 |
|  | E3P1 | 142.13 | < 0.0001 |  | **F1** | **F2** | **F3** | **F4** | **F5** |
|  |  |  |  | **F1** | -3.31 | **5.89** | -2.84 | -3.38 | **4.08** |
|  |  |  |  | **F2** | **3.20** | -2.53 | **3.59** | -2.08 | -2.17 |
|  |  |  |  | **F3** | -2.04 | 0.12 | -1.70 | **5.19** | -1.82 |
|  |  |  |  | **F4** | -1.40 | -1.50 | **2.96** | -2.50 | **2.70** |
|  |  |  |  | **F5** | **3.13** | -2.29 | -1.80 | **3.44** | -2.93 |
|  | E3P2 | 110.77 | < 0.0001 |  | **F1** | **F2** | **F3** | **F4** | **F5** |
|  |  |  |  | **F1** | 0.08 | 0.95 | -0.49 | -1.08 | 0.56 |
|  |  |  |  | **F2** | **1.97** | -2.75 | **4.92** | -1.41 | -1.47 |
|  |  |  |  | **F3** | -1.02 | 1.41 | -2.80 | **3.81** | -2.25 |
|  |  |  |  | **F4** | -1.18 | -2.92 | 1.65 | -3.93 | **6.32** |
|  |  |  |  | **F5** | 0.41 | **3.35** | -2.93 | **2.22** | -3.32 |
| **8** | E2P1 | 231.10 | < 0.0001 |  | **F1** | **F2** | **F3** | **F4** | **F5** |
|  |  |  |  | **F1** | -1.73 | -1.32 | -2.87 | -3.15 | **8.45** |
|  |  |  |  | **F2** | **4.61** | -1.86 | **2.97** | -2.47 | -1.81 |
|  |  |  |  | **F3** | -1.04 | **8.36** | -3.56 | 0.23 | -3.63 |
|  |  |  |  | **F4** | -1.65 | -2.54 | **7.28** | -3.81 | 0.09 |
|  |  |  |  | **F5** | 0.80 | -2.99 | -3.15 | **8.75** | -3.63 |
|  | E2P2 | 190.93 | < 0.0001 |  | **F1** | **F2** | **F3** | **F4** | **F5** |
|  |  |  |  | **F1** | 0.22 | -0.86 | -1.34 | -1.71 | **4.88** |
|  |  |  |  | **F2** | 0.78 | -2.17 | **3.90** | -2.41 | 0.05 |
|  |  |  |  | **F3** | -0.64 | **8.73** | -5.13 | -0.49 | -2.88 |
|  |  |  |  | **F4** | -0.60 | -3.41 | **6.35** | -4.59 | 2.05 |
|  |  |  |  | **F5** | 0.58 | -3.52 | -3.46 | **8.49** | -2.32 |
|  | E3P1 | 201.89 | < 0.0001 |  | **F1** | **F2** | **F3** | **F4** | **F5** |
|  |  |  |  | **F1** | -3.70 | 1.71 | -0.62 | -3.03 | **5.56** |
|  |  |  |  | **F2** | **7.63** | -2.90 | **2.04** | -3.19 | -3.31 |
|  |  |  |  | **F3** | -2.70 | **5.29** | -1.92 | **2.53** | -3.36 |
|  |  |  |  | **F4** | -2.85 | -1.89 | **2.78** | -2.65 | **5.33** |
|  |  |  |  | **F5** | 1.62 | -2.70 | -1.80 | **5.83** | -3.57 |
|  | E3P2 | 156.24 | < 0.0001 |  | **F1** | **F2** | **F3** | **F4** | **F5** |
|  |  |  |  | **F1** | -2.40 | 0.73 | -0.24 | -2.44 | **4.59** |
|  |  |  |  | **F2** | 1.53 | 1.76 | 0.44 | -1.38 | -1.35 |
|  |  |  |  | **F3** | -2.00 | 1.16 | -0.88 | **6.80** | -4.99 |
|  |  |  |  | **F4** | -3.26 | -1.30 | **2.26** | -4.51 | **7.29** |
|  |  |  |  | **F5** | **6.19** | -1.38 | -1.38 | -0.07 | -4.59 |
| **9** | E2P1 | 141.59 | < 0.0001 |  | **F1** | **F2** | **F3** | **F4** | **F5** |
|  |  |  |  | **F1** | -2.68 | 1.69 | -1.47 | -1.98 | **4.57** |
|  |  |  |  | **F2** | **3.73** | -2.54 | **4.64** | -2.47 | -3.6 |
|  |  |  |  | **F3** | -2.90 | 3.65 | -2.70 | **5.13** | -2.49 |
|  |  |  |  | **F4** | -1.56 | -0.94 | 0.61 | -2.77 | **4.39** |
|  |  |  |  | **F5** | **3.20** | -1.65 | -1.75 | **2.50** | -2.51 |
|  | E2P2 | 102.45 | < 0.0001 |  | **F1** | **F2** | **F3** | **F4** | **F5** |
|  |  |  |  | **F1** | -2.77 | 0.49 | 0.72 | -1.56 | **3.42** |
|  |  |  |  | **F2** | 0.94 | 0.53 | 0.77 | -0.39 | -1.38 |
|  |  |  |  | **F3** | -1.09 | 1.50 | -1.94 | **4.99** | -3.08 |
|  |  |  |  | **F4** | -2.45 | -1.12 | **2.31** | -3.87 | **5.01** |
|  |  |  |  | **F5** | **5.35** | -1.04 | -1.52 | 0.62 | -4.18 |
|  | E3P1 | 361.85 | < 0.0001 |  | **F1** | **F2** | **F3** | **F4** | **F5** |
|  |  |  |  | **F1** | -3.19 | **4.55** | -1.15 | -3.08 | **2.44** |
|  |  |  |  | **F2** | **4.54** | -2.59 | 2.26 | -2.10 | -1.31 |
|  |  |  |  | **F3** | -3.99 | **3.00** | -1.61 | **6.23** | -4.06 |
|  |  |  |  | **F4** | -2.82 | -2.70 | **2.32** | -3.14 | **7.08** |
|  |  |  |  | **F5** | **5.98** | -2.63 | -1.39 | 1.18 | -3.66 |
|  | E3P2 | 116.66 | < 0.0001 |  | **F1** | **F2** | **F3** | **F4** | **F5** |
|  |  |  |  | **F1** | -3.73 | 0.04 | -1.50 | -1.39 | **5.61** |
|  |  |  |  | **F2** | **4.85** | -3.29 | 3.06 | -1.44 | -2.87 |
|  |  |  |  | **F3** | -3.84 | **5.57** | -1.79 | **2.23** | -1.66 |
|  |  |  |  | **F4** | -1.16 | -1.44 | 0.70 | -0.19 | **2.15** |
|  |  |  |  | **F5** | **4.20** | -1.87 | -0.20 | 0.70 | -2.77 |
| **10** | E2P1 | 243.13 | < 0.0001 |  | **F1** | **F2** | **F3** | **F4** | **F5** |
|  |  |  |  | **F1** | -3.19 | 0.53 | -1.76 | -2.98 | **7.52** |
|  |  |  |  | **F2** | **8.24** | -2.18 | 0.01 | -3.11 | -3.65 |
|  |  |  |  | **F3** | -3.12 | **5.88** | -3.02 | **3.45** | -1.68 |
|  |  |  |  | **F4** | -3.89 | -1.74 | **7.44** | -3.64 | 1.43 |
|  |  |  |  | **F5** | 1.56 | -1.96 | -3.34 | **6.70** | -3.34 |
|  | E2P2 | 147.87 | < 0.0001 |  | **F1** | **F2** | **F3** | **F4** | **F5** |
|  |  |  |  | **F1** | -2.85 | 0.21 | -1.11 | -2.63 | **6.07** |
|  |  |  |  | **F2** | 1.33 | 0.29 | **2.14** | -1.54 | -1.70 |
|  |  |  |  | **F3** | 1.23 | 0.55 | -2.72 | **4.98** | -4.03 |
|  |  |  |  | **F4** | -3.16 | -1.24 | **4.68** | -4.27 | **3.87** |
|  |  |  |  | **F5** | **3.72** | 0.36 | -2.48 | **2.79** | -4.34 |
|  | E3P1 | 252.23 | < 0.0001 |  | **F1** | **F2** | **F3** | **F4** | **F5** |
|  |  |  |  | **F1** | -3.31 | **6.30** | -3.04 | -3.51 | **2.56** |
|  |  |  |  | **F2** | **3.77** | -3.19 | **6.34** | -2.71 | -3.14 |
|  |  |  |  | **F3** | -2.99 | **2.73** | -2.73 | **6.40** | -3.66 |
|  |  |  |  | **F4** | -2.42 | -3.59 | **2.15** | -3.06 | **6.81** |
|  |  |  |  | **F5** | **5.86** | -3.12 | -2.25 | **3.06** | -3.07 |
|  | E3P2 | 138.81 | < 0.0001 |  | **F1** | **F2** | **F3** | **F4** | **F5** |
|  |  |  |  | **F1** | -2.42 | 1.28 | -1.15 | -2.30 | **4.51** |
|  |  |  |  | **F2** | 0.34 | 0.45 | 0.49 | -0.78 | -0.26 |
|  |  |  |  | **F3** | -1.24 | 1.26 | -2.87 | **5.16** | -2.07 |
|  |  |  |  | **F4** | -3.49 | -1.69 | **6.38** | -3.97 | **2.41** |
|  |  |  |  | **F5** | **6.78** | -0.86 | -2.99 | 1.44 | -4.28 |
|  | E2P1  11 | 172.37 | < 0.0001 |  | **F1** | **F2** | **F3** | **F4** | **F5** |
|  |  |  |  | **F1** | -3.59 | **3.45** | -3.23 | -2.22 | **5.29** |
|  |  |  |  | **F2** | **5.61** | -2.72 | 1.27 | -2.43 | -2.15 |
|  |  |  |  | **F3** | -2.28 | **3.99** | -2.47 | **4.04** | -2.47 |
|  |  |  |  | **F4** | -2.74 | -2.66 | **6.09** | -2.38 | 1.42 |
|  |  |  |  | **F5** | **3.30** | -2.16 | -1.82 | **3.77** | -2.79 |
|  | E2P2 | 119.27 | < 0.0001 |  | **F1** | **F2** | **F3** | **F4** | **F5** |
|  |  |  |  | **F1** | -2.40 | 1.07 | 0.01 | -1.69 | **3.35** |
|  |  |  |  | **F2** | 0.52 | 0.34 | 1.18 | -1.22 | -0.48 |
|  |  |  |  | **F3** | -1.69 | 0.88 | -2.67 | **5.49** | -2.15 |
|  |  |  |  | **F4** | -3.10 | -1.31 | **4.77** | -3.93 | **3.65** |
|  |  |  |  | **F5** | **6.43** | -0.64 | -2.79 | 0.58 | -4.01 |
|  | E3P1 | 185.81 | < 0.0001 |  | **F1** | **F2** | **F3** | **F4** | **F5** |
|  |  |  |  | **F1** | -2.56 | **5.47** | -2.06 | -2.15 | 1.47 |
|  |  |  |  | **F2** | **2.85** | -3.20 | **3.72** | -2.77 | -1.03 |
|  |  |  |  | **F3** | -2.96 | **4.49** | -3.78 | **3.78** | -1.50 |
|  |  |  |  | **F4** | -2.60 | -2.96 | **5.45** | -2.95 | **3.50** |
|  |  |  |  | **F5** | **5.17** | -3.33 | -3.39 | **3.57** | -2.21 |
|  | E3P2 | 127.68 | < 0.0001 |  | **F1** | **F2** | **F3** | **F4** | **F5** |
|  |  |  |  | **F1** | -4.11 | **5.60** | -1.81 | -2.54 | **2.30** |
|  |  |  |  | **F2** | **3.93** | -3.30 | **3.81** | -1.51 | -2.16 |
|  |  |  |  | **F3** | -1.40 | 0.86 | -0.07 | 1.61 | -0.89 |
|  |  |  |  | **F4** | -1.74 | -0.17 | 0.52 | -2.52 | **4.23** |
|  |  |  |  | **F5** | **2.78** | -2.75 | -2.07 | **5.12** | -3.54 |
| **12** | E2P1 | 168.08 | < 0.0001 |  | **F1** | **F2** | **F3** | **F4** | **F5** |
|  |  |  |  | **F1** | -2.13 | **5.05** | -3.87 | -3.61 | **4.51** |
|  |  |  |  | **F2** | 1.22 | -2.56 | **4.42** | -1.02 | -1.92 |
|  |  |  |  | **F3** | -1.30 | **2.22** | -2.65 | **3.57** | -2.30 |
|  |  |  |  | **F4** | -1.66 | -2.43 | **5.44** | -3.29 | 1.56 |
|  |  |  |  | **F5** | **4.13** | -2.77 | -3.02 | **5.06** | -2.64 |
|  | E2P2 | 140.71 | < 0.0001 |  | **F1** | **F2** | **F3** | **F4** | **F5** |
|  |  |  |  | **F1** | 0.35 | **2.40** | -1.71 | -1.84 | 1.32 |
|  |  |  |  | **F2** | 0.97 | -2.20 | **3.96** | -1.54 | -0.88 |
|  |  |  |  | **F3** | -0.98 | **5.36** | -4.22 | 1.90 | -2.89 |
|  |  |  |  | **F4** | -1.05 | -3.62 | **4.85** | -4.62 | **4.68** |
|  |  |  |  | **F5** | 1.20 | -1.69 | -2.90 | **5.68** | -2.24 |
|  | E3P1 | 171.24 | < 0.0001 |  | **F1** | **F2** | **F3** | **F4** | **F5** |
|  |  |  |  | **F1** | -2.65 | **3.71** | -3.38 | -2.63 | **5.52** |
|  |  |  |  | **F2** | **3.15** | -2.65 | **5.02** | -3.15 | -2.39 |
|  |  |  |  | **F3** | -0.95 | **3.02** | -3.01 | **3.33** | -2.42 |
|  |  |  |  | **F4** | -1.50 | -2.24 | **4.82** | -3.06 | 1.63 |
|  |  |  |  | **F5** | 1.92 | -1.97 | -3.30 | **5.80** | -2.67 |
|  | E3P2 | 99.78 | < 0.0001 |  | **F1** | **F2** | **F3** | **F4** | **F5** |
|  |  |  |  | **F1** | -2.80 | **5.045** | -0.72 | -1.80 | 0.098 |
|  |  |  |  | **F2** | 1.401 | -3.35 | **5.499** | -2.98 | -1.05 |
|  |  |  |  | **F3** | -0.47 | -0.41 | -3.75 | **5.932** | -1.60 |
|  |  |  |  | **F4** | 1.834 | -1.93 | -0.12 | -1.99 | **3.412** |
|  |  |  |  | **F5** | 0.782 | 0.214 | -1.55 | 0.865 | -0.18 |
